# Cancer incidence and mortality in Poland in 2019

**DOI:** 10.1038/s41598-022-14779-6

**Published:** 2022-06-27

**Authors:** Joanna Didkowska, Urszula Wojciechowska, Irmina Maria Michalek, Florentino Luciano Caetano dos Santos

**Affiliations:** Polish National Cancer Registry, Maria Sklodowska-Curie National Research Institute of Oncology, ul. Wawelska 15B, 02-093 Warsaw, Poland

**Keywords:** Cancer, Statistics, Epidemiology, Cancer, Cancer screening, Cancer epidemiology

## Abstract

The purpose of this paper is to offer the most important epidemiological indicators of malignant neoplasms in Poland for the year 2019. In 2019, the Polish National Cancer Registry received information on almost 171.2 thousand new cancer cases and 100.3 thousand cancer deaths. The most common male cancers were prostate (20.6%), lung (16.1%), colon (6.8%), bladder (6.4%), and rectal (4.2%) cancers. Age-standardized incidence rates were at 118 per 100,000 for prostate, 89 for lung, 40 for colon, 38 for bladder, and 23 for the rectum. The most prevalent female cancers encompassed breast (22.9%), lung (9.9%), corpus uteri (7.0%), colon (5.9%), and ovary (4.3%). Age-standardized incidence rate was at 95 per 100,000 for breast cancer, 40 for lung 40, 29 for corpus uteri, 24 for colon, and 18 for ovarian cancer. The five leading causes of male cancer deaths were cancer of the lung (27.4%), prostate (10.3%), colon (8.0%), bladder (5.8%), and stomach (5.7%). Age-standardized mortality rates were 100 per 100,000 for lung, 46 for prostate, 32 for colon, 24 for urinary bladder, and 22 for stomach cancer. Most female deaths due to cancer were caused by lung (17.9%), breast (15.1%), colon (7.7%), ovary (6.0%), pancreas (5.7%), and corpus uteri (4.0%) cancers. Age-standardized mortality rates were 39 per 100,000 for lung, 33 for breast, 17 for colon, 13 for ovarian, 13 for pancreatic, and 9 for corpus uteri cancer.

## Introduction

Cancer constitutes a growing health, social, and economic problem. This problem's scale is reflected in the increasing number of incident cases and deaths globally. According to GLOBOCAN estimates, only in 2020, around 19.3 million new cancer cases were diagnosed worldwide, and almost 10.0 million individuals died due to cancer. Moreover, the global cancer burden is expected to be 28.4 million cases per year in two decades, denoting an almost 50% rise^1^.

Also, in Poland, cancer has a considerable impact on public health, influencing healthcare expenditures, burdening cancer survivors and their families, and remaining one of the leading causes of death. In 2009, Poland spent EUR 1.05 billion on oncology, in 2018—EUR 2.1 billion, and in 2019—EUR 2.3 billion. In 2020 the expenditure was estimated at EUR 2.4 billion. These amounts included services provided under the so-called oncology package, but also part of the services: clinical oncology, oncological surgery, oncological gynecology and hematology, chemotherapy, radiotherapy, and drug programs related to oncological treatment^2^. Noteworthy, these values did not encompass indirect costs of cancer-related absenteeism in the workplace. Finally, cancer was the second leading cause of death in Poland over the last decade, accounting for almost one-fourth of all male and female deaths^3^.

In 2015, the United Nations Members States adopted the 2030 Agenda for Sustainable Development, with several Sustainable Development Goals, including improving health by reducing premature mortality due to non-communicable diseases by 2030. Effective national cancer control plans must address the rising cancer burden to meet this goal. The Polish parliament adopted a new National Strategy for Oncology (NSO) 2020–2030 in 2020 to improve evidence-based prevention, population screening programs, appropriate treatment, and palliative care. The overarching goal of NSO is to increase the percentage of people surviving five years after completion of oncological therapy. The initiative also encourages cancer surveillance through cancer registries and attempts to boost cancer survivability.

Polish National Cancer Registry (PLCR) collects, stores, and manages the data on all cancer cases diagnosed within the Polish population. The PLCR offers important insight into cancer epidemiology in Central Europe and covers a population of 38.0 million individuals^4^, representing the most extensive cancer registry with full-population coverage in European Union. Since 1979, the PLCR has published an annual statistical bulletin entitled „Cancer in Poland”. This cyclical publication is addressed to everyone interested in the epidemiology of malignant neoplasms in Poland. The current edition of the report pertains to the cancer situation in 2019 and presents the most up-to-date data and information on cancer epidemiology in Poland^5^.

The overarching goal of this article is to present a synopsis of the most important cancer epidemiological indicators in Poland for the year 2019.

## Materials and methods

### Source of data and identification of cancer cases

Data were obtained from the PLCR^6^, a non-profit national institution responsible for statistical and epidemiological cancer research in Poland (population 38.0 million in June 2019^4^). The registry covers practically all incident cancer cases diagnosed in the Polish population since 1965. Data are actively collected from hospitals, healthcare practitioners, and palliative care centers. Reporting cancer to the PLCR is mandatory. All notifications are submitted by physicians and coded according to the International Statistical Classification of Diseases and Related Health Problems, Tenth Edition (ICD-10). At the PLCR, all cases are afterward verified by qualified coders based on histopathological/cytological/cytometry exam results and additionally coded applying the International Classification of Diseases for Oncology, Third Edition (ICD-O-3). Finally, the records are passed through specific tools to validate entries based on the valid causes of death in relation to the sex and age of the deceased. The PLCR collection system is based on a unique Polish personal identification number (PESEL), which avoids double coding for the same patient. The reported data on incident cases is still burdened with some under-registration. Hence, in this publication, the term "incidence" should be understood as a registered incidence.

We identified all Polish patients diagnosed with malignant neoplasms registered in the PLCR database between the 1st of January 2019 and the 31st of December 2019 and collected as of the 3rd of December 2021. Vital status was verified based on yearly controls of registered patients' vital status in the PESEL database. Since PLCR is a population-based registry that uses PESEL for registration and employs the PESEL database for yearly follow-up, the likelihood of loss to follow-up is minimal. Individuals who emigrated permanently from Poland and had no legal tie with the country may account for a meager portion of under-reported deaths.

### Statistical analysis

Crude annual incidence rates were defined as the number of new cases diagnosed per 100,000 person-years. Annual crude mortality rates were defined as the number of new deaths among cancer patients per 100,000 person-years. In the denominator, we applied the mid-year population, defined as the population's size on the 31st of June of the respective year. To enable comparison with other populations, we performed direct age-standardization of incidence and mortality for the revised European Standard Population (ESP2013). Since data are presented per annum, and the denominator is the middle-year population, we communicate the metrics per 100,000 inhabitants in the Results section for simplicity of presentation.

Fifteen-year (2005–2019) incidence trends and annual percent change (APC), with 95% confidence intervals (CI), were calculated for seven cancer sites, i.e., colorectum (ICD-10 C18–C21), lung (C33–C34), female breast (C50), cervix (C53), ovary (C56), prostate (C61), and skin melanoma (C43). Joinpoint regression was applied, and the best-fitting model was selected with permutations tests, with an overall significance level at 0.05. Rates were considered to decrease if APC < 0 and 95% CI did not contain zero, and to increase if APC > 0 and 95% CI did not contain zero; otherwise, rates were considered stable.

Spatial cluster analysis of the age-standardized (ESP2013) incidence and mortality rates (ASR) was conducted based on the Getis-Ord local statistics Gi*^7^ at the powiat level (corresponding to county or district in other countries—local administrative unit-1 level). Such a granular analyses was conducted due to the high age-standardized mortality rates in northern Poland. The identification of statistically significant hot spots (high Gi* value) allows the identification of above-average mortality areas. The spatial neighborhood of each powiat was defined as all its neighboring powiats. The test for independence was conducted using 1000 conditional permutation simulations.

Colormaps used Jenks natural breaks classification method (maximization of variance between classes and minimization within classes). Age-standardized mortality rates’ Getis-Ord local statistics Gi* colormaps were centered at 0 (white, no statistically significant z-scores) with high mortality spatial clusters depicted in red color and low mortality spatial clusters depicted in blue color.

Joinpoint analysis and graphs were performed using the Joinpoint Regression software (version 4.3.1.0, National Cancer Institute, Bethesda, MD, USA). Statistical and spatial analysis, choropleth maps, and age-standardized incidence and mortality maps were produced using RStudio Version 1.4.1103 (R Foundation for Statistical Computing, Vienna, Austria).

### Ethical standards

According to Polish legislation, individual-level data from the PLCR can be used for statistics in aggregate form and scientific purposes. The PLCR obeys strict regulations to secure the complete confidentiality and protection of the individuals.

## Results

### Overall national analysis

**Figure 1:**
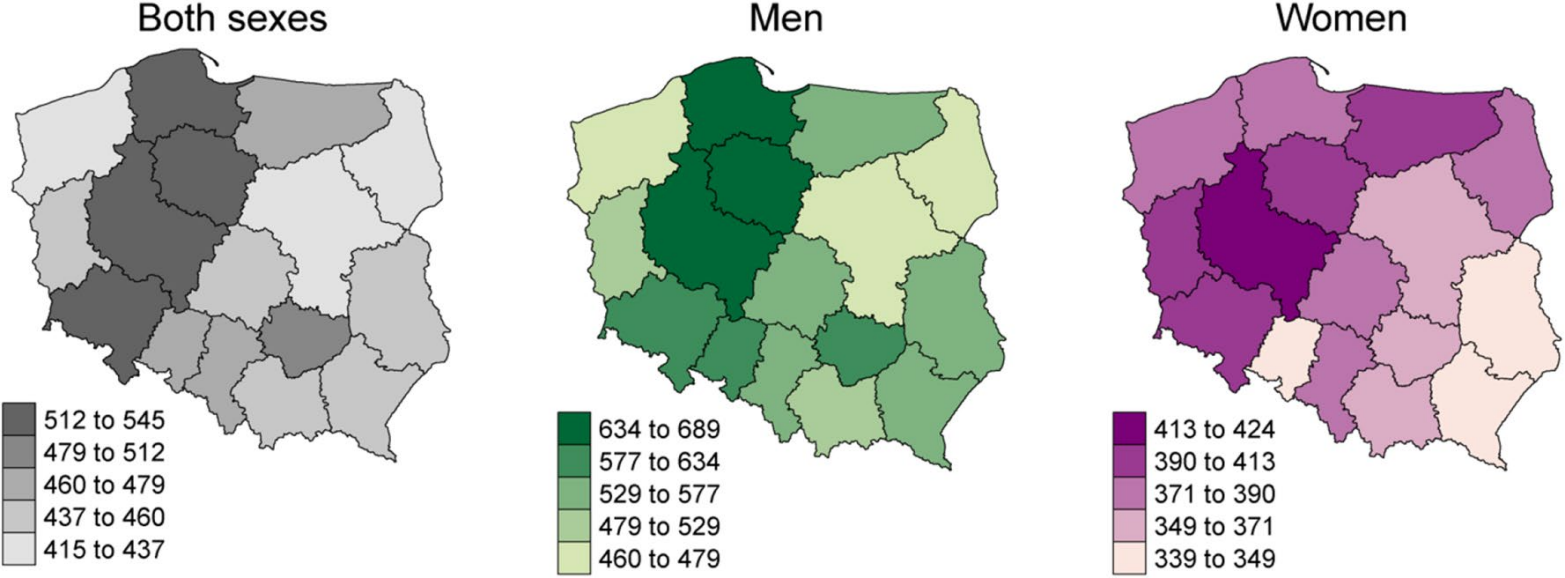
Age-standardized (ESP2013) incidence rates per 100,000 for all cancer sites aggregated, by sex—Poland, 2019.

**Figure 2:**
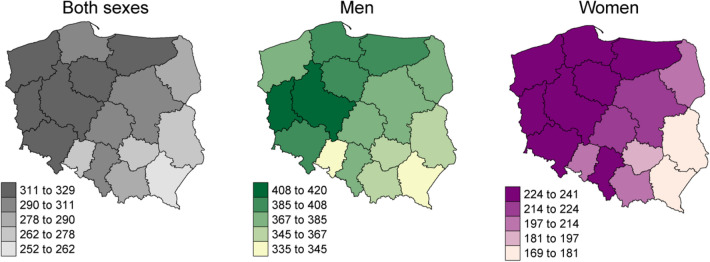
Age-standardized (ESP2013) mortality rates per 100,000 for all cancer sites aggregated by sex—Poland, 2019.

**Figure 3:**
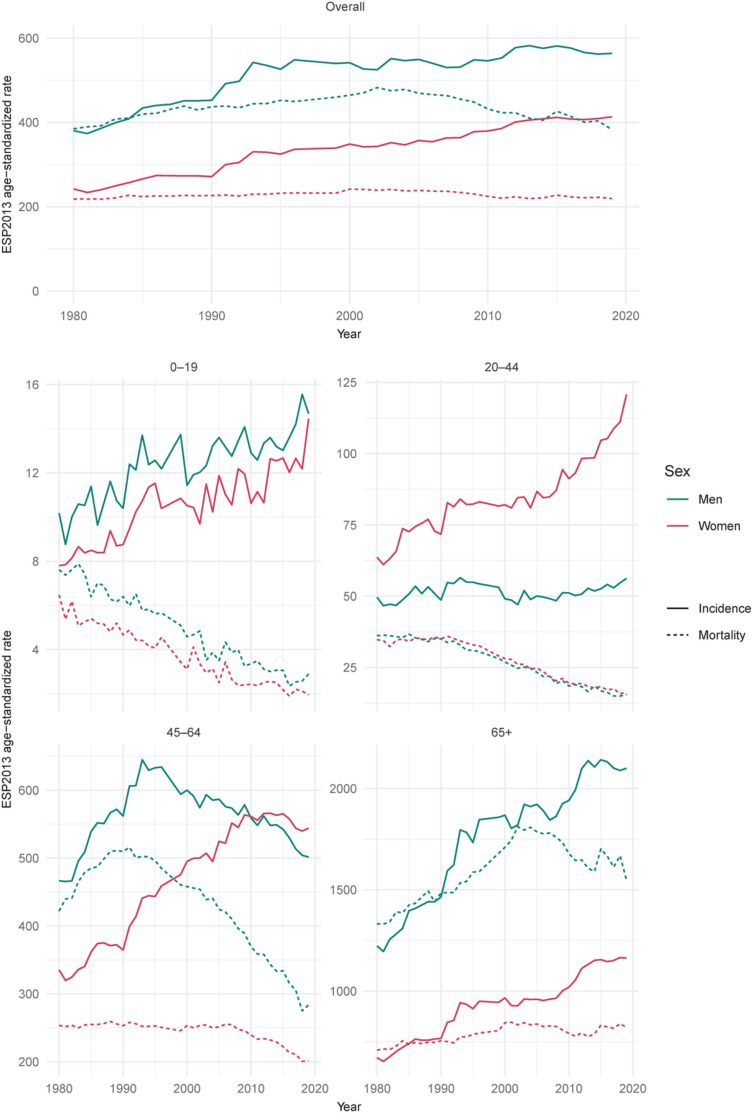
Age-standardized (ESP2013) incidence and mortality rates’ per 100,000 time trends, overall and by age group, by sex—Poland, 2019.

**Figure 4:**
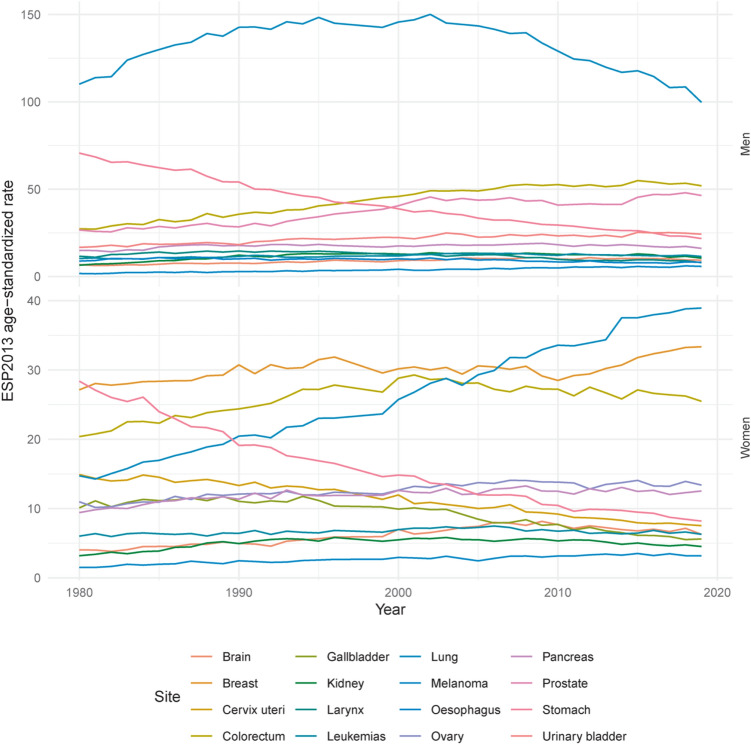
Age-standardized (ESP2013) mortality rates per 100,000 for the most common cancer sites, by sex—Poland, 2019.

In 2019, in Poland, there were 171,218 incident cancer cases (85,559 among men and 85,659 among women) and 100,324 deaths due to cancer (54,370 among men and 45,954 among women). The age- and sex-standardized (ESP2013) incidence and mortality rates were higher in northwest than in the southeast regions of Poland, independently of sex (Figs. 1, 2). The overall age- and sex-standardized incidence rate for primary cancers was 446 per 100,000 inhabitants (564 per 100,000 men and 413 per 100,000 women; Fig. 3). The overall age- and sex-standardized mortality rate was 383 per 100,000 inhabitants (383 per 100,000 men and 219 per 100,000 women; Fig. 4).

**Figure 5:**
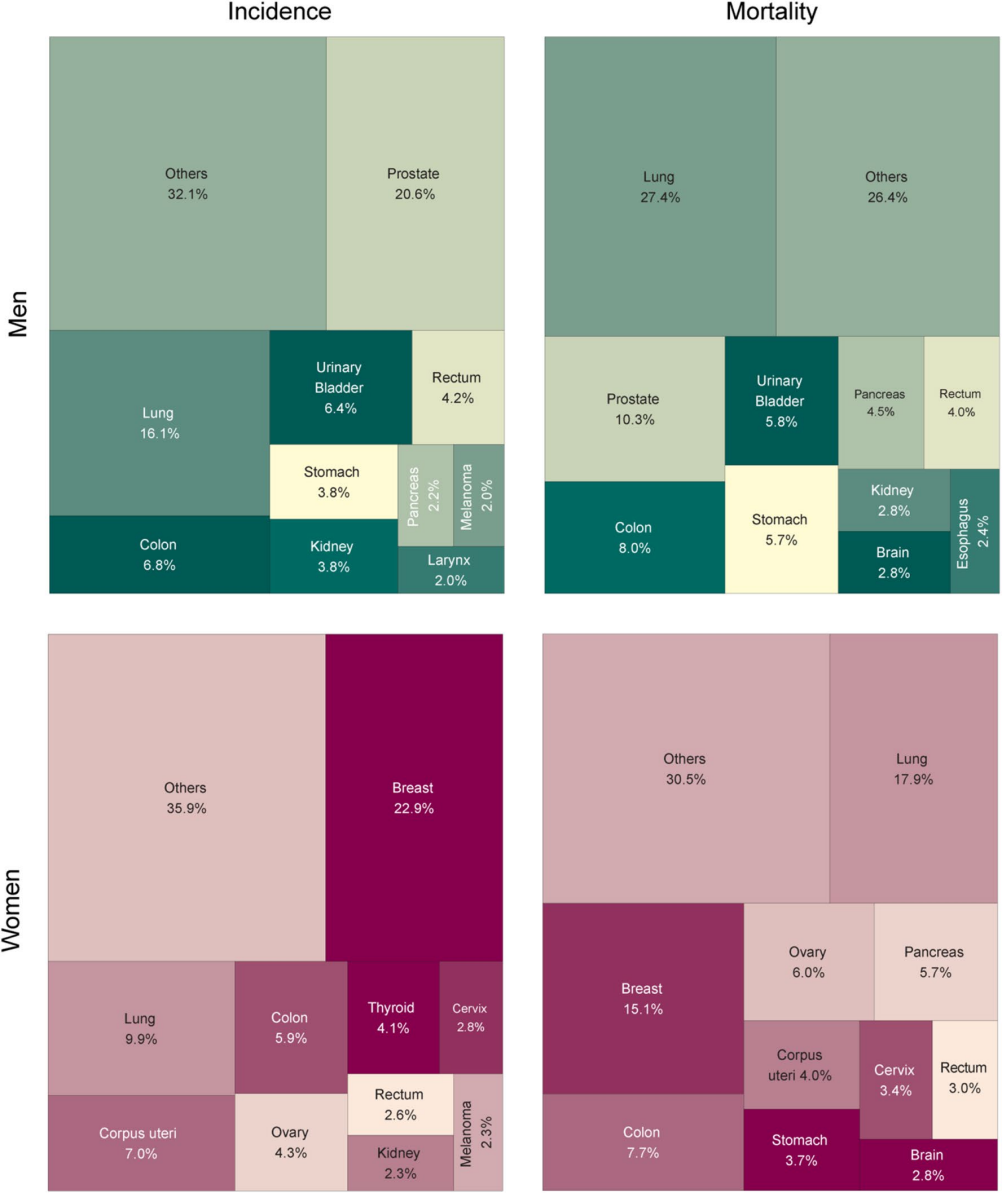
Structure of cancer incidence and mortality, by sex—Poland, 2019.

The five most common cancers in men encompassed prostate (20.6%), lung (16.1%), colon (6.8%), urinary bladder (6.4%), and rectum (4.2%; Fig. 5). Age-standardized incidence rates were at 118 per 100,000 men for prostate cancer, 89 for lung cancer, 40 for colon cancer, 38 for bladder cancer, and 23 for the rectal cancer. The five most common cancers in women pertained to the breast (22.9%), lung (9.9%), corpus uteri (7.0%), colon (5.9%), and ovary (4.3%; Fig. 5). The age-standardized incidence rate was 95 per 100,000 for breast cancer, 40 for lung cancer, 29 for corpus uteri cancer, 24 for colon cancer, and 18 for ovarian cancer.

The five leading causes of mortality among men were cancer of the lung (27.4%), prostate (10.3%), colon (8.0%), bladder (5.8%), and stomach (5.7%). Age-standardized mortality rates were 100 per 100,000 for lung cancer, 46 for prostate cancer, 32 for colon cancer, 24 for urinary bladder cancer, and 22 for stomach cancer (Fig. 4). Noteworthy, lung cancer mortality was almost two-folds higher than cancer of the prostate. Among women, most cancer deaths were due to cancer of the lung (17.9%), breast (15.1%), colon (7.7%), ovary (6.0%), pancreas (5.7%), and corpus uteri (4.0%). Age-standardized mortality rates were 39 per 100,000 for lung cancer, 33 for breast cancer, 17 for colon cancer, 13 for ovarian cancer, 13 for pancreatic cancer, and 9 for corpus uteri cancer (Fig. 4).

### Incidence time-trends

Regarding incidence time-trends, in the last 15 years, the highest APC was observed for prostate cancer (APC + 4.3%, 95% CI + 3.8% to + 4.8%). The second highest APC was for melanoma, with an upward trend, independent on sex (men APC + 3.5%, 95% CI + 2.7% to + 4.3%; women APC + 3.6%, 95% CI + 2.5% to + 4.7%). For female breast cancer, the incidence has been increasing since 2009, around + 1.2% per year (95% CI + 0.7% to + 1.6%). Lung cancer time trends differed by sex. While men presented a downward trend since 2012 (APC − 3.3%, 95% CI − 3.9 to − 2.6%), since 2014 an upward trend was observed for women (APC + 1.2%, 95% CI + 0.1 to + 2.3%). In the last ten years, colorectal cancer incidence has been decreasing in both sexes—among men since 2015, with an APC at − 1.7% (95% CI − 3.3 to − 0.1%) and among women since 2014 with APC at − 1.5% (95% CI − 2.3 to − 0.6%). For cervical cancer, the incidence has been systematically decreasing since 2005 (APC − 3.2%, 95% CI − 3.8 to − 2.7%). Ovarian cancer incidence has been stable between 2005 and 2019.

### Age group analysis

In 2019, childhood (0–19 years of age) cancers were responsible for 6.6% of all deaths among boys and 7.3% among girls. The most prevalent tumors of children were leukemias, lymphomas, and brain tumors, accounting for over 60% of all cases in this age group. A similar distribution was observed in mortality, independently of sex.

Among young adults (20–44 years of age), the age-standardized incidence rate of female cancers was more than double that of male cancers (120 vs. 56 per 100,000). The most common male cancers in this group pertained to the testicle (23% of cases, 8% of deaths), colorectum (7% of cases, 9% of deaths), and skin melanoma (6% of cases, 5% of deaths). Young men’s mortality was primarily from brain tumors (14%). Among young women, the most common cancer sites were breast (28% of cases, 27% of deaths), cervix (5% of cases, 10% of deaths), ovary (5% of cases, 8% of deaths), and colon (3% of cases, 6% of deaths).

The age-standardized cancer incidence rate for middle-aged men (45–64 years of age) was 502 per 100,000 and for women it was 544 per 100,000. The most common male cancers in this group pertained to lung (17% of cases, 30% of deaths), prostate (17% of cases, 4% of deaths), colon (12% of cases, 11% of deaths), bladder (5% of cases, 4% of deaths), and stomach (4% of cases, 6% of deaths). The most prevalent female cancers affected breast (29% of cases, 18% of deaths), lung (9% of cases, 21% of deaths), colon (8% of cases, 9% of deaths), ovary (6% of cases, 9% of deaths), and corpus uteri (9% of cases, 3% of deaths).

**Figure 6:**
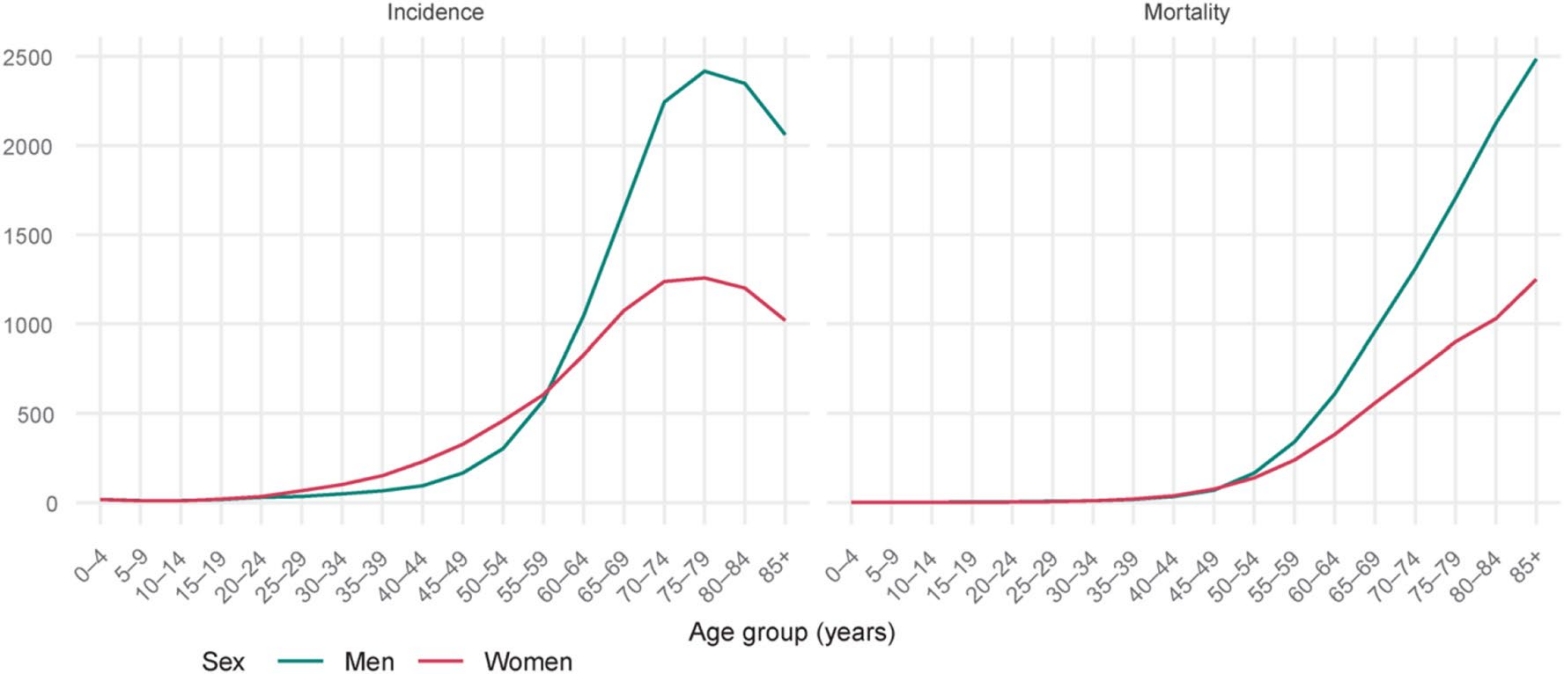
Cancer incidence and mortality rates per 100,000, by age group—Poland, 2019.

In the oldest age-group (> 65 years of age), the age-standardized incidence rate of female cancers was more than double that of male cancers than female cancers (2100 vs. 1163 per 100,000; Fig. 6). In this age group, the most common male cancer sites encompassed prostate (24% of cases, 13% of deaths), lung (17% of cases, 27% of deaths), colon (13% of cases, 14% of deaths), bladder (7% of cases, 7% of deaths), and stomach (4% of cases, 6% of deaths). Among women, they included breast (19% of cases, 14% of deaths), colon (12% of cases, 13% of deaths), lung (12% of cases, 17% of deaths), corpus uteri (7% of cases, 5% of deaths), and ovary (4% of cases, 5% of deaths).

### Spatial clustering of mortality rates

**Figure 7:**
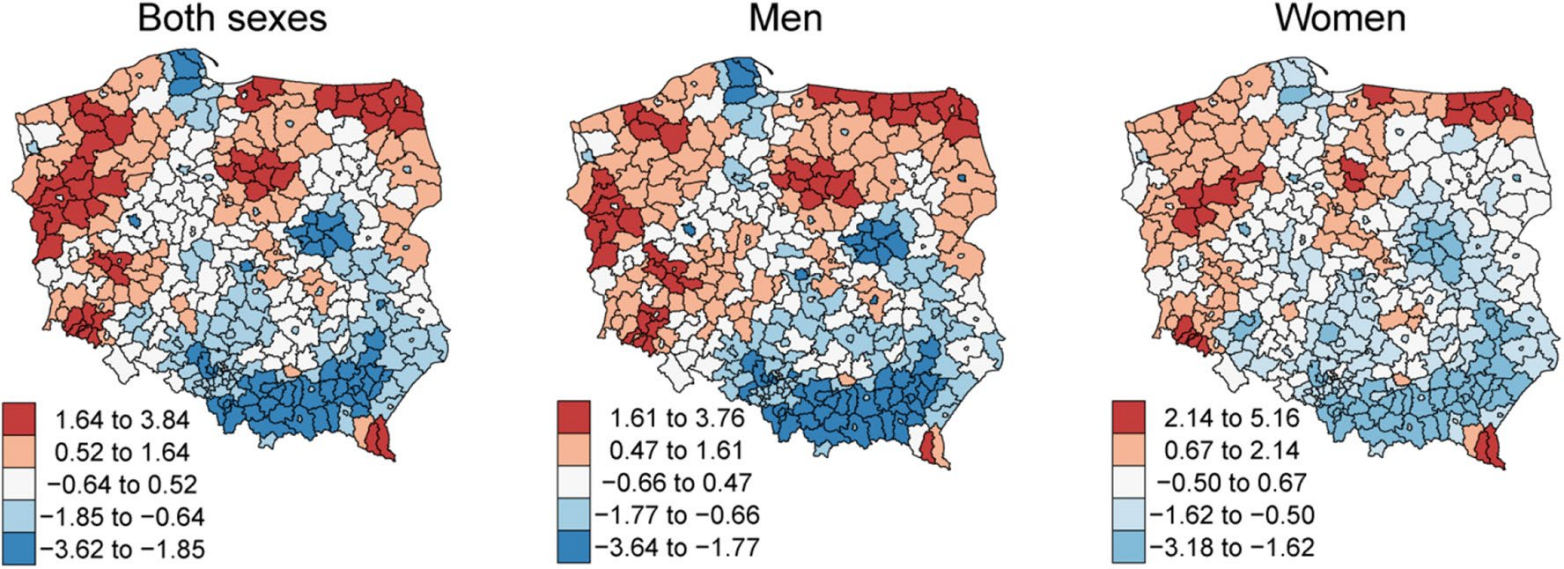
Clusters of age-standardized mortality rates (expressed as Getis-Ord G_i_^*^) for all cancer sites aggregated, powiat level—Poland, 2019.

There was a preponderance of high age-standardized mortality rates’ clusters in northern Poland compared with the southern regions in spatial clustering analysis. This pattern was observed for both sexes, except for one high-value cluster of female mortality rates in the southernmost regions of the Podkarpackie voivodship (southeast Poland; Fig. 7). The age-standardized mortality due to cancer differed between regions, especially in the case of lung cancer, with a clear split between north-western and south-eastern parts of the country. Although lung and colorectal cancer were the biggest influencers in male and female mortality rates’ spatial clustering patterns, the decrease in Gi* from northwest to the southeast was also noticeable for prostate and female breast cancers. No clear regional clustering pattern was observed for male and female pancreatic cancer (Figure S1).

## Discussion

### Main findings of the report

This study provides thorough and up-to-date cancer epidemiology data for Poland. According to the PLCR data, in 2019, there were 171,218 incident cancer cases and 100,324 deaths due to cancer. In 2019, age- and sex-standardized (ESP2013) incidence and mortality rates' spatial patterns concurred with the previously published PLCR annual reports, with higher rates in northwest Poland and a gradual decrease towards the southeast regions, independently of sex.

The annual number of incident cases has quadrupled between 1965 and 2019 (from around 38 thousand to more than 171 thousand per year)^5^. Also, the crude number of deaths due to cancer increased in this time (from about 39 thousand to 100 thousand per year). The increase in the incidence and mortality due to cancer observed in Poland in the last 50 years can be attributed to changes in the population's age structure^8^ and the increase in the registered incidence by the mid-1980s, as a result of the improvement of cancer registration and coverage.

Since 2016, the structure of cancer incidence among Polish men has been similar to that observed in other countries with a very high Human Development Index, namely with the domination of prostate and lung cancer^9–13^. In most voivodships (corresponding to a province in other countries; 14 out of 16), prostate cancer was the most common male cancer, followed by lung cancer. Lung was the leading cancer topography in the two other voivodships. Noteworthy, lung cancer mortality was almost two-folds higher than for prostate cancer. In all 16 voivodships, the highest mortality rate was attributed to lung cancer. The second most common cause of cancer-related death was colorectal (14 out of 16 voivodships) or prostate cancer (2 out of 16 voivodships).

The structure of cancer incidence among Polish women was dominated by breast cancer, with incidence twice as high as for lung cancer. In all voivodships (16 out of 16) breast was the leading cancer site. However, in 14 of the 16 voivodships, lung cancer was the leading cause of cancer-related deaths. The second and third positions were shared by breast and colorectal cancer. Although in the last 15 years, the lung cancer has become the main contributor to female cancer-related mortality (a position occupied from the mid-1970s to the mid-2000s by breast cancer), the breast cancer mortality rate trend has changed back to upward in the last decade, and breast cancer may in the future again become the leading cause of female death due to cancer.

The most obvious finding to emerge from this report is that, since the beginning of the twenty-first century, the mortality due to male lung cancer has been decreasing, and female lung cancer has been increasing. These changes can be attributed to changes in smoking patterns over time—decline in smoking rates by men and increase by women^14,15^. To assure a decrease in tobacco-related female lung cancer mortality rates, more efforts are needed. Not only must women-targeted smoking cessation services be available, but policymakers should also enforce increasing the excise duty on tobacco products and outlawing menthol and slim cigarettes. The Tobacco Products Directive (2014/40/EU) has already implemented some of these concepts. However, this regulation has only been in effect for a short time and has had no impact on mortality figures. Nonetheless, Polish policymakers should make effective implementation of the Directive a priority because it has the potential to reduce lung cancer mortality among Polish women further.

### COVID-19 pandemia, the war in Ukraine and their potential consequences

The present manuscript represents a snapshot of the state of cancer in Poland in 2019, before the COVID-19 pandemic (the first case in Poland in March 2020^16^). The pandemia has compelled temporary limitations in admittance to planned therapies, delayed diagnosis, and cancer screening routines^17,18^. As presented in several previous international studies, the lower affluence on cancer screening programs has increased the incidence of advanced-stage cancers, mortality rates, and years of life lost^19,20^. This scenario is also expected to be observed in Poland from 2020 onwards. Future works should also investigate the influence of the COVID-19 pandemics on national cancer survival.

Furthermore, due to the hosting of over 3.5 million Ukrainian refugees in Poland (state for 1st of June 2022), it is expected that the national cancer incidence, prevalence, and survival will shift due to the differences in these metrics in the Ukrainian population^21^. Nonetheless, the consequences of the pandemia and refugee influxes will take time to be perceptible.

### Completeness and quality of cancer registration

The condition for a sound assessment of cancer epidemiology is the high reliability of the data, both in terms of completeness and case coding quality. In the last 30 years, the fraction of morphologically verified cancer cases reported to PLCR, for all cancers aggregated, increased from 51 to 91%. In 2019, around 91% of the reported male cancer cases and 92% of female cases were confirmed by a pathological examination. At the same time, the mortality/incidence ratio decreased from 1.0 to 0.6. Both metrics reflect a noticeable quality improvement of cancer reporting and registration in Poland.

### Strengths and limitations of the report

The report's key strength is the considerable study population and close-to-complete nationwide coverage, which ensures the study's representativity and generalizability. Furthermore, because the state finances the Polish healthcare system, everyone has equal access to diagnostic and treatment procedures, regardless of socioeconomic position; therefore, we do not expect underreporting owing to a lack of diagnosis caused by the high cost of healthcare.

It is important to remember that cancer registries keep track of cancer cases, not patients. Because a patient may have many primary malignancies, they may appear in a registry database more than once. Two cases are included when a person has two primary malignancies in two locations, e.g., lung and breast.

Our study has the constraint that, despite regulations requiring timely reporting of new cases, late reports are received for various reasons. Providers may overlook deadlines; institutions may find other cases, and death certificates reveal unreported cases. For investigators and registry staff dedicated to accurateness, precision, consistency, and timeliness, these scenarios are challenging.

### Implications of the study

The results presented in this study enable an evaluation of the current oncological strategy and its effectiveness on an annual basis (annual bulletin) and a comparison between Poland and other countries' health metrics. Following the previous reflections, the present study can affect national cancer control policies both in the near and long-term, such as predicting the extra future strain on the Polish health system by considering the effect of last years' pandemia and extraordinary migratory influx.

## Conclusions

In 2019, the Polish National Cancer Registry received information on almost 171 thousand new cancer cases and 100 thousand cancer deaths. During the last two decades, the overall age-standardized cancer incidence increased consistently, the mortality remains relatively stable.

In 2019, the most common male cancers pertained to prostate, lung, colon, urinary bladder, and rectum. The leading causes of mortality in men were lung, prostate, colon, bladder, and stomach cancer. The most prevalent female cancers encompassed breast, lung, corpus uteri, colon, and ovary cancer. Most female cancer deaths were due to lung, breast, colon, ovary, pancreas, and corpus uteri cancer.

Cancer constitutes a significant health problem in Poland, where its incidence and mortality trends are determined by the population pyramid and changes in the exposure to carcinogens, especially tobacco smoking. The impact of smoking prevalence is especially evident in the female population. In 2019, the number of female lung cancer deaths exceeded the number of breast cancer deaths.

## Supplementary Information

Supplementary Information.

## Data Availability

The data analyzed in this study was obtained from the PLCR and is available upon reasonable request by contacting the PLCR at krn@pib-nio.pl, and subject to the ethical approvals in place and material transfer agreements. Summarized and aggregated data can be obtained from the website of the PLCR (http://onkologia.org.pl/raporty/), which also provides options to conduct cross-sectional analyses deploying data collected during 1999–2019.
