# Supplementary material for: Cancer incidence and mortality in Poland in 2019
Source: Sci Rep. 2022 Jun 27;12:10875. doi: 10.1038/s41598-022-14779-6 (PMC9237124; doi:10.1038/s41598-022-14779-6)
Supplement: Supplementary file 1 — Supplementary Information. [file 41598_2022_14779_MOESM1_ESM.pdf]

## Supplementary material – Figures

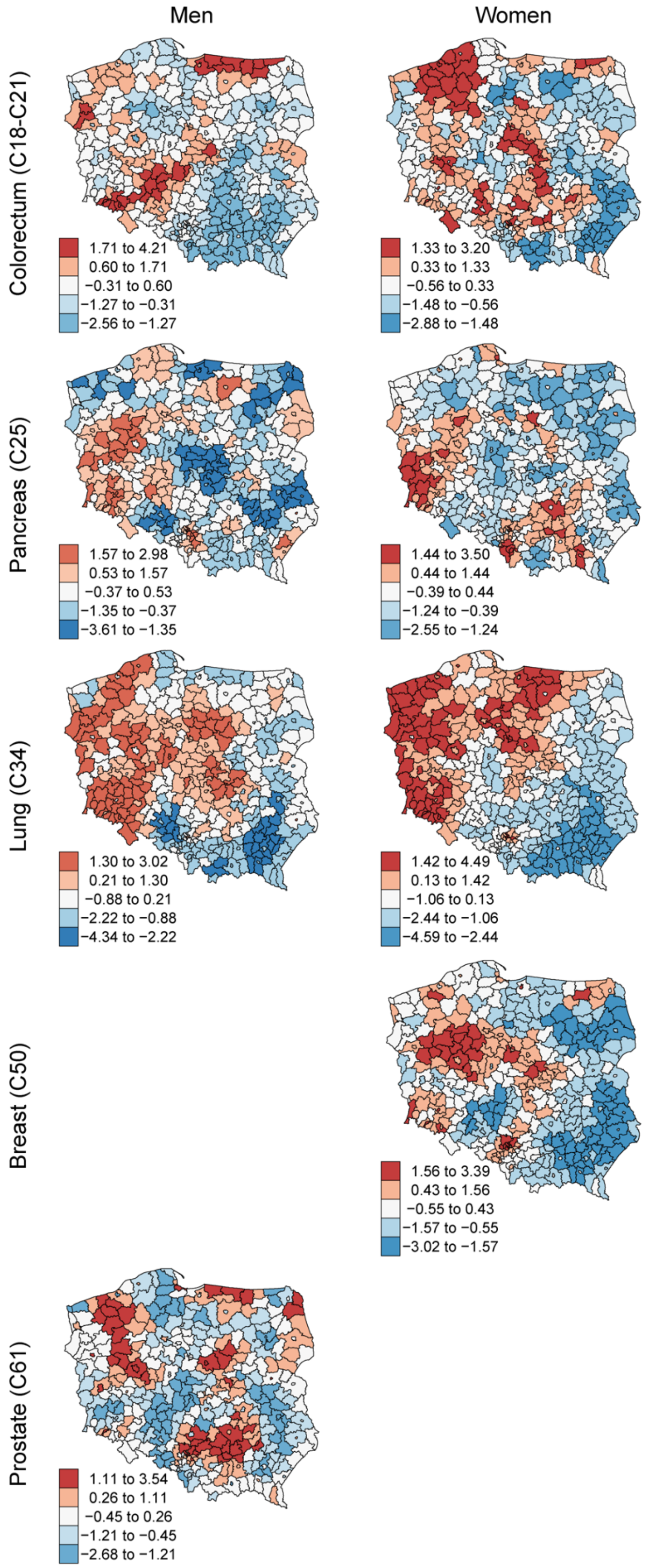


**Figure S1.** Getis-Ord Gi* for selected high mortality cancer-sites, by sex, powiat level - Poland, 2019.
